# Sleep quality in children with hepatic glycogen storage diseases, a prospective observational pilot study

**DOI:** 10.1002/jmd2.12462

**Published:** 2024-12-10

**Authors:** Lucas Agnoletto, Moya Vandeleur, Mary White, Anne‐Marie Adams, Rebecca Halligan, Heidi Peters

**Affiliations:** ^1^ Murdoch Children's Research Institute Melbourne Victoria Australia; ^2^ Department of Paediatrics University of Melbourne Melbourne Victoria Australia; ^3^ Department of Respiratory and Sleep Medicine Royal Children's Hospital Melbourne Victoria Australia; ^4^ Department of Endocrinology and Diabetes Royal Children's Hospital Melbourne Victoria Australia; ^5^ Melbourne School of Global and Population Health, University of Melbourne Melbourne Victoria Australia; ^6^ Department of Metabolic Medicine Royal Children's Hospital Melbourne Victoria Australia; ^7^ Department of Inherited Metabolic Diseases Evelina London Children's Hospital London UK

**Keywords:** continuous glucose monitoring (CGM), Glycaemic control, hepatic glycogen storage disorders, Paediatric, sleep, sleep disruption

## Abstract

**Background:**

Hepatic glycogen storage diseases (GSDs) are characterised by enzyme defects affecting liver glycogen metabolism, where carbohydrate supplementation to prevent overnight hypoglycaemia is common. Concerns around sleep quality in hepatic GSDs relate to emerging evidence that overnight dysglycaemia impacts sleep quality.

**Methods:**

This prospective observational study reported sleep quality and duration in children with hepatic GSDs over 7 days utilising: actigraphy (Actiwatch 2 by Phillips Respironics), sleep diaries, proxy reported age‐appropriate sleep and quality‐of‐life (QoL) questionnaires, in the context of nocturnal glycaemic profiles continuous glucose monitor (CGM, Dexcom G6) and nocturnal dietary management strategies. Significant hypo‐ and hyperglycaemia were defined as ≥1% of sleep diary documented nocturnal period, recording <3.5 and >10.0 mmol/L, respectively.

**Results:**

Seven children with hepatic GSD (aged 1–17 years) participated. Objective sleep quality was poor, with actigraphy demonstrating that no child achieved the minimum sleep duration recommended for age. Subjective sleep quality was also poor, with 4/5 documenting significant daytime sleepiness and 6/6 reporting poor sleep hygiene. Children prescribed overnight bolus feeds (OBF) (*n* = 2) recorded shorter sleep duration compared to other nocturnal management strategies. Parent‐reported QoL suggested poor disease‐related QoL outcomes for this cohort.

**Conclusion:**

Objective and subjective sleep disturbances and reduced QoL are common within our sample of children with hepatic GSD. From our observations these outcomes may be linked to nutritional overnight interventions, especially OBFs, rather than overnight glucose levels. Consideration of the impacts of overnight feeding strategies on sleep quality and QoL in children with hepatic GSD should inform future management strategies.

**Synopsis**

This paper aims to evaluate sleep quality within a cohort of children with hepatic glycogen storage diseases in the context of overnight glucose levels and nutritional interventions.

## 1 INTRODUCTION

Glycogen storage diseases (GSDs) are rare genetic disorders defined by enzyme deficiencies in the glycogen metabolism pathways affecting the liver and muscles.^1^, ^2^ The subtypes of GSD are distinguished by specific enzyme defects and variations in affected tissues.^3^ Disease presentations involve hypoglycaemia with short fasting intervals and hepatomegaly.^1^ Treatment aims are to maintain euglycemia (blood glucose [BG] between 4.0 and 7.8 mmol/L) to optimise growth and prevent secondary consequences.^4^ Nocturnal management of overnight glycemia may consist of a single dose of long‐acting uncooked cornstarch (UCCS) and potentially supplemented with additional overnight bolus feeds (OBFs) or continuous overnight gastric feedings (COGFs).^3^, ^5^, ^6^ More intensive feeding therapies are often required in GSD type 1, owing to the combined defect in glycogenolysis and gluconeogenesis.^2^

Adequate sleep quality and duration are imperative for optimal growth and development.^7^ Healthy sleep encompasses adequate age‐dependent duration, consistent sleep patterns, and satisfactory sleep quality.^8^ Research shows that 30–40% of infants and school‐aged children experience sleep problems, which are associated with poor quality of life(QOL) outcomes, especially if left untreated.^9^, ^10^, ^11^ Children diagnosed with chronic diseases, are at higher risk of developing multifaceted sleep disturbances, such as insomnia or sleep‐disordered breathing (SDB).^12^, ^13^ Polysomnography (PSG) is the diagnostic gold standard for sleep assessment, however actigraphy is a validated, more easily administered tool, that when used with a sleep diary, can measure sleep patterns and quality over consecutive nights at home.^14^

Whilst there is ample research into the effects of chronic disease on sleep quality, to our knowledge there are no published studies specifically assessing sleep quality in children with hepatic GSDs. Poor sleep quality has been described in limited detail in the hepatic GSD population.^15^, ^16^ One study collected actigraphy data from a small cohort (GSD‐Ia adults) and observed poor sleep quality.^15^ The other retrospectively analysed patient medical history records, briefly documenting the potential presence of sleep disturbances.^16^ However, there is an emerging literature base evaluating sleep in children with type 1 diabetes (T1D).^17^, ^18^, ^19^ T1D and hepatic GSDs share some similarities in their fluctuation of glycaemic profiles, resulting in hypo/hyper‐glycaemic episodes despite treatment and the requirement for nocturnal disease management.^2^, ^20^ Current T1D literature identifies that rapid changes to nocturnal BG levels are associated with poorer sleep quality and we postulate that GSD populations may be at risk for similar sleep disturbances.^2^, ^20^, ^21^ This study aims to evaluate sleep quality in a paediatric cohort of hepatic GSD individuals and to observe any potential trends between sleep quality and glucose levels.

## 2 METHODS

### 2.1 Study subjects

The primary objective of this prospective observational study was to document sleep quality in children with hepatic GSDs over seven nights. Secondary objectives assessed the relationship between objective and subjective sleep quality, QOL and overnight BG. Participants were recruited from the Royal Children's Hospital (RCH), Melbourne metabolic medicine outpatient clinic from March to August 2022 and had confirmed diagnosis of hepatic GSD by a paediatric Metabolic Medicine specialist.

### 2.2 Sleep assessment

#### 2.2.1 Actigraphy

Children wore an actigraph (Actiwatch‐2, MiniMitter, Philips Healthcare, Andover, MA) continuously on their nondominant wrist for 7 days. Data was downloaded onto commercially available software (Actiware Software Package, version 5.5, MiniMitter; Philips Healthcare) for analysis. Measures of interest included, total sleep time (TST; number of minutes scored as sleep in the sleep period), sleep onset latency (SOL; number of minutes to fall asleep), wake after sleep onset (WASO; number of minutes scored as wake in the sleep period), sleep efficiency (SE; sleep duration/sleep period*100) and movement fragmentation index (MFI; a sleep fragmentation index, measuring sleep disturbance).

A recent concept analysis amalgamated objective sleep data from actigraphy studies and determined standard clinical cut‐off scores for school‐aged children.^22^ For TST, the American Academy of Sleep Medicine has published standardised clinical cut‐off for recommended sleep times.^23^ For MFI, a recent study determined the MFI for a healthy population, which was used as this study's comparison point.^24^ Actigraphy data was validated using a parental/guardian proxy sleep diary.

#### 2.2.2 Sleep questionnaires

Parents and children were asked to complete validated, age‐dependent questionnaires regarding their child's sleep behaviours:

**Adolescent/children's sleep hygiene scale (A/CSHS):** The A/CSHS is a 24‐item self‐report questionnaire assessing sleep‐facilitating and sleep‐inhibiting behaviours in adolescents between 12 and 18 years of age or children between the ages of 2 and 11 years old.^25^ It assesses six different domains of sleep, with an overall score of 3.8, representing the lowest quintile from the original study population.^25^

**Obstructive sleep apnoea (OSA)‐18:** The OSA‐18 is a parent report proxy used to determine health‐related‐QOL (HRQOL) impacts of OSA in children between the ages of 6 months and 18 years.^26^ It asses five sleep disturbance domains, with scores >80 representing a clinically significant impact of OSA upon HRQOL.^26^

**Paediatric daytime sleepiness scale (PDSS):** The PDSS is an 8‐item self‐report questionnaire that measures daytime sleepiness in children aged 5–17 years old.^27^ The original study assessing healthy children reported mean total scores of 15.3 ± 6.2. Scores greater than this indicate significant daytime sleepiness.^27^

**Sleep disturbance scale for children (SDSC):** The SDSC is a 26‐item Likert‐type rating scale completed by parents assessing sleep disturbances in children 3–18 years old.^28^ A total cut‐off score of >39 was determined to identify children who experienced disturbed sleep quality when validating against polysomnography and actigraphy.^28^, ^29^

### 2.3 QOL questionnaires

**Paediatric quality of life inventory 4.0 generic core scale (PedsQL):** The PedsQL is a parent‐reported measure of a child's HRQOL, validated for children 5–18 years. The 23 items encompass 4 domains: Physical, Emotional, Social, and School Functioning. The Total Scale score is the mean sum of all the items in each scale. The original paper determined a minimum cut‐off score of 69.7 as a marker of individuals who would experience HRQOL concerns due to their condition.^30^

### 2.4 CGM

**FIGURE 1:**
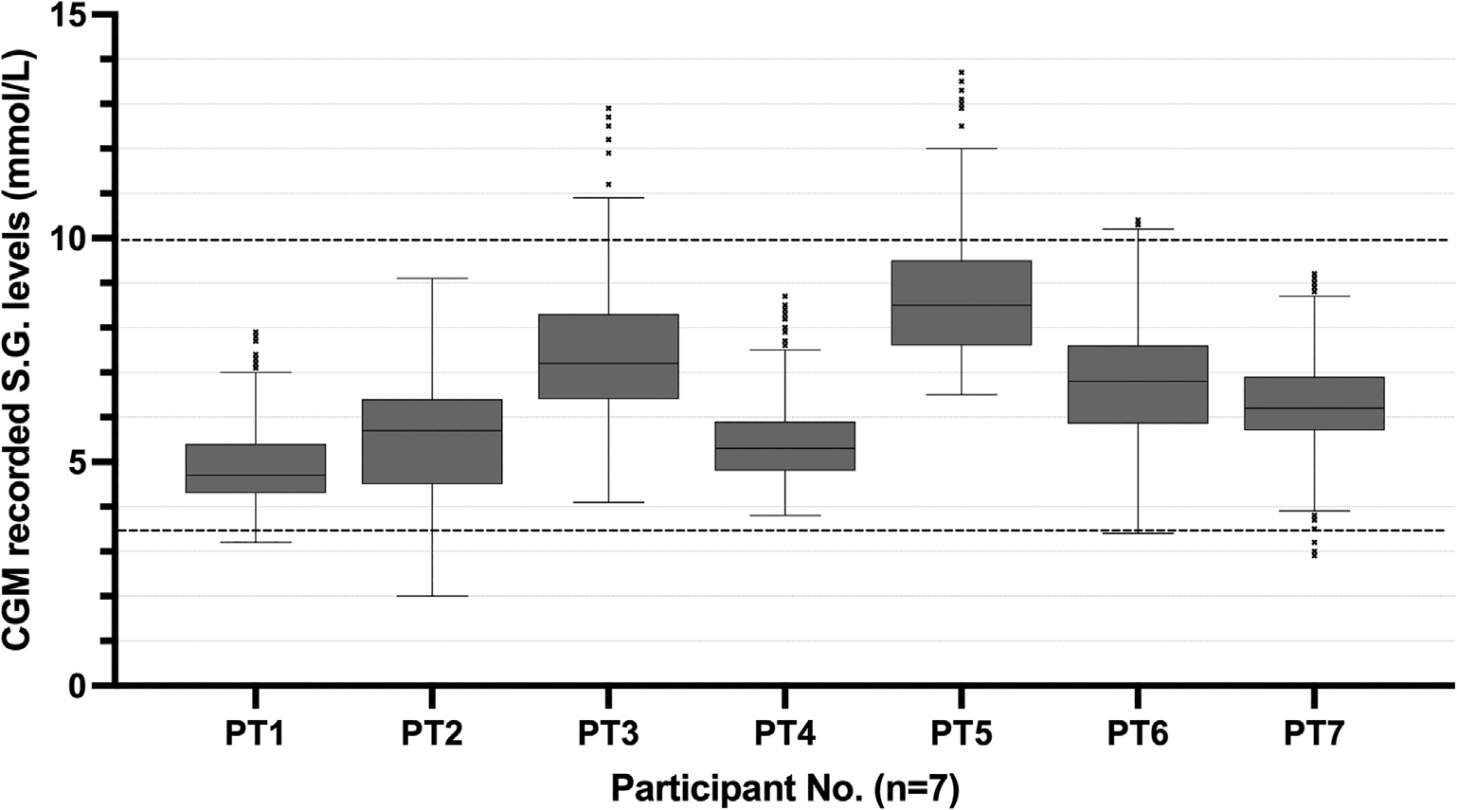
Continuous glucose monitor (CGM) recorded subcutaneous glucose (S.G.) Data During Seven Nights of Sleep, *n* = 7. Participant number (Participant No.) *n* = 7. Box plot parameters; are the 25th percentile (lower hinge of the box) and the 7th percentile (upper hinge of the box), with the horizontal line within the box representing the median. The whiskers represent the lower and upper adjacent values, 25th percentile – 1.5 × IQR and 75th percentile +1.5 × IQR, respectively. The small ‘x's represent outlier values that fall outside the lower and upper adjacent values. Participant No.: Participant number. CGM, continuous glucose monitor; S.G, subcutaneous glucose. Dashed line at *y* = 3.5 represents hypoglycaemic threshold used in this study. Dashed line at *y* = 10.0 represents hyperglycaemic threshold used in this study.

Participant's subcutaneous glucose (SG) levels were measured using a CGM (Dexcom G6) a clinically validated tool for measuring SG levels.^17^ This device was blinded so only the study team had access to SG data, and therefor participants were instructed to undertake standard disease management protocols. The metabolic parameters recorded were nocturnal mean and standard deviation (SD) of SG (mmol/L), and the percentage recordings below 3.5 and above 10.0 mmol/L(%) during sleep. Scores <1% were not clinically relevant. The glycaemic parameters selected are in line with markers used in by the metabolic medicine team at RCH in their clinical care and therefor are of clinical interest. In conjunction with the CGM, a food diary of overnight dietary management was completed by the parent/guardian which assisted the analysis and validation of the CGMs data (Figure 1).

## 3 RESULTS

### 3.1 Demographic characteristics

All patients/carers of children with hepatic GSD at The RCH's Metabolic Medicine clinic were approached to participate in the study. Seven participants (7/23, 30.4%, 5 male), were recruited, ranging from 1 to 17 years old and all participants completed seven nights of CGM and actigraphy evaluation (Table 1). Of the seven participants, six individuals were prescribed a patient specific, pre‐bedtime dose of a UCCS, Glycosade. Of those receiving Glycosade four participants received additional nocturnal nutrition, two via a COGF and two via OBFs (Table 1). The Glycosade is administered orally and acts as an initial buffer against hypoglycaemia. The COGFs were delivered via a G‐tube and functioned as continuous administration of carbohydrates, whereas of OBFs required the participants to be awoken for additional Glycosade feeds during the night.

**TABLE 1:** Patient Demographics, Nocturnal Dietary Management Strategies and Individual Measurement Outcomes.

	Screening tool	Outcome measure	Clinically significant cut‐off scores	1	2	3	4	5	6	7
Age				1	13	2	15	17	6	12
GSD subtype				Ib	Ia	VI	IXc	III	Ia	IXa
Genotype				SLC37A4 c.625G > C (p.Gly209Arg) homozygous	n/a	pygl gain (exons 6–13) copy number = 4	*PHKG2*: c.247C > T (p.Gln83X) – likely pathogenic, and c.96‐11G > A	n/a	*PHKG2*: c.247C > T (p.Gln83X) – likely pathogenic, and c.96‐11G > A	PHKA2 p.G300D hemizygous
UCCS use before sleep				Yes	Yes	Yes	No	Yes	Yes	Yes
Nocturnal dietary intervention (in addition to UCCS)				OBF	COGF	OBF	None	None	COFG	None
Sleep quality	Actigraphy	TST (age‐appropriate recommendation) (min)		**389.3** [720]	**426.7** [480]	**512.2** [660]	**441.4** [480]	**390.7** [480]	**459.0** [540]	**436.9** [540]
		SOL (delayed onset of sleep) (min)	≥45 min	**78.1**	17.3	6.2	17.4	25.1	11.6	6.4
		WASO (time awake during nocturnal sleep period) (min)	≥41 min	**75.1**	**49.3**	**75.2**	29.4	38.1	**50.4**	**68.0**
		SE (time asleep during nocturnal sleep period) (%), *n*	<74%	**60.3**	82.8	75.2	81.9	79.9	80.4	84.5
		MfI (movement during sleep period)	≥26.8	**28.0**	25.1	**47.9**	10.7	22.8	**33.9**	**30.4**
	PDSS	Daytime sleepiness	>15.3		14		**22**	**21**	**21**	**17**
	SDSC	Disturbed sleep	>39		29		39	33	**40**	37
	OSA‐18	Presence of possible OSA	>80	28	20	38	21	22	31	27
	A/CSHS	Sleep hygiene	<3.8		**2.4**	**2.6**	**2.2**	**2.3**	**2.4**	**2.3**
Quality of life		Peds‐QL (QOL impact due to chronic disease) scores above clinical cut‐off, *n*	>69.7		77.2	84.5	89.1	73.9	79.4	84.8
Glycaemic profiles		Time SG <3.5 mmol/L	>1.0	0.62	**10.64**	0	0	0	0.25	0.55
		Time SG > 10.0 mmol/L	>1.0	0	0	**3.25**	0	**16.48**	0.63	0

*Note*: TST cut off determined from standardised clinical cut‐off (23). MFI clinical cut off score determined from MFI determination within health population. (24). All other objective sleep data analysed from standardised clinical cut‐offs (22). Scores in bold are above or below standard clinical cut‐offs. Genotype variations were not available for participants who had not completed genetic analysis PT2 and PT5.

Abbreviations: ASHS, adolescent's sleep hygiene scale; COGF: continuous overnight gastric feed; CSHS, children's sleep hygiene scale; OBF: overnight bolus feed; Peds‐QL: Paediatric Quality of Life Inventory 4.0 Generic Core Scale; PDSS: paediatric daytime sleepiness scale; SDSC, sleep disturbance scale for children; SE, sleep efficiency; SG, subcutaneous glucose; SOL, sleep onset latency; TST, total sleep time; UCCS, uncooked cornstarch; WASO, wake after sleep onset.

### 3.2 Actigraphy

Seven participants completed at‐home actigraphy. All children demonstrated a shorter mean TST duration than recommended for age, according to the American Academy of Sleep Medicine clinical practice guidelines.^23^ 5/7 children (71.4%) recorded scores outside the clinically recommended ranges in at least one of the objective sleep disturbance measurements (Table 1).

### 3.3 Sleep and QOL questionnaires

All participants completed at least one subjective sleep quality questionnaire. 4/5 children who completed the PDSS, 4/4 children completed the ASHS and 6/7 of those completing Peds‐QL reported clinically significant scores in the respective measures. All children completed the OSA‐18 questionnaire and recorded scores below the clinically significant cut‐off value.

### 3.4 CGM data

All seven participants completed CGM analysis for the seven nights during sleep (Table 1). One individual (PT2) recorded clinically significant levels of hypoglycaemia, 10.64% of readings (Table 1). Two individuals (PT3 and PT5) recorded clinically significant hyperglycaemia, 3.25% and 16.48% of recordings respectively (Table 1).

## 4 DISCUSSION

In this small cohort of children with hepatic GSD we found a high prevalence of poor objective sleep quality. None of the children met the minimum recommended age‐dependent sleep duration.^23^ WASO and MFI, a measure of total minutes spent awake during the nocturnal sleep period and sleep fragmentation; was elevated in the majority of participants (5/7). For one participant (PT1), all recorded variables fell outside the recommended ranges, however they were 18 months old, when sleep is highly variable.^31^

Self‐reported subjective sleep quality was also sub‐optimal, where elevated daytime sleepiness in 4/5 (PDSS score) and poor sleep hygiene was noted for all 6 child participants who completed the age‐specific surveys. Interestingly only one parent reported significant sleep disturbance with an elevated SDSC total score. This disparity may be a contributing factor to the under‐recognition, and resultant under‐diagnosis of sleep disorders in children with hepatic GSD, highlighting an area for future studies.

One previous study directly measured sleep in individuals with hepatic GSD.^15^ This study explored the impact of nocturnal euglycemia, via the use of Glycosade and therefore less nighttime interruptions, would have on sleep quality within a cohort of adults (*n* = 8), diagnosed with GSD Ia. Baseline objective sleep characteristics, measured by actigraphy, found normal sleep quantity, sleep efficiency and an elevated WASO within this cohort.^15^ The subjective sleep quality survey, measured by the Pittsburgh Sleep Quality index (PSQI) found that at baseline, scores of this cohort were significantly above the clinically significant cutoff. However, after the use of Glycosade, there was significant improvement in subjective sleep quality, with the domains that were most affected being; day dysfunction due to sleepiness scale and overall sleep quality scale.^15^ There was no improvement in other domains of sleep. Comparison of objective sleep data showed conflicting results, with all individuals in our cohort recording poor sleep quantity (TST). However, both studies recorded elevated WASO and normal SE. When analysing these conflicting outcomes, there are differences in study design that must be taken into consideration. In individuals with GSD Ia, fasting tolerance increases with age. As this study was conducted in adults, disease pathophysiology would be markedly different between the two cohorts.^32^ And so, no individuals from the prior study required nocturnal interventions, which may account for the differences in TST.^15^ The use of different subjective sleep questionnaires and conflicting study cohort demographics, means that assessing the implications of the different outcomes is difficult.

With CGM we were able to assess the association between hypo‐ or hyperglycaemic episodes and sleep quality. Past literature in T1D has suggested that the arousal threshold is potentially altered by hypoglycaemic events.^33^, ^34^, ^35^ PT2's CGM documented clinically significant time within hypoglycaemic range (Table 1). This individual also recorded elevated WASO, an indicator of arousal (Table 1). This suggests that the same mechanism may have affected this individual in our study. Yet four other individuals also recorded elevated WASO without corresponding hypoglycaemia, and therefor there may be another factor influencing WASO. Due to this small sample size, further research in larger cohorts is required to determine whether the physiological responses to these episodes are similar or different to T1D individuals. The two individuals that recorded clinically significant time in hyperglycaemia, recorded contrasting levels of sleep quality. PT3, recorded decreased TST and elevated WASO and MFI whereas PT5 who recorded higher levels of hyperglycaemia, recorded inadequate TST, but a normal WASO, MFI and SE. However, PT3 was prescribed a OBFs, whereas PT5 had no overnight intervention further suggesting the impact of nocturnal intervention on sleep quality.

Nocturnal management strategies were observed to be a potential influence on sleep quality within this population. Four of five children who recorded elevated WASO, were receiving an overnight intervention (Table 1). The two individuals on OBFs, recorded the smallest TST, when compared to their age‐recommended minimums (Table 1).^23^ This preliminary data suggests that the nature and presence of nocturnal interventions may be influential on sleep quality. Past studies have demonstrated that food intake during the nocturnal period negatively affects sleep quality within healthy individuals.^36^ There are multiple potential mechanisms in which the different overnight interventions influence sleep architecture and quality. OBFs, produce larger glycaemic variability, when compared to COGFs, and which may impact the central nervous system's regulation of sleep–wake cycles.^36^ This may be a contributing factor as to why there are differences seen in sleep quality between the different overnight intervention groups, with the OBF group (*n* = 2) recording 8 out of 10 abnormal objective sleep quality results compared to the 5 within the COGF group (*n* = 2). However it is clear the need for further research to explore these initial observations.

There was a few limitations that may have impacted the observations seen within this study. This study involved the introduction of a CGM, which is not normally standard care for this patient population. Whilst the participant was blinded from the CGM data, site irritation and general device discomfort may have contributed to poorer sleep quality, however this is unlikely as after insertion CGMs are generally well tolerated. This study also did not track specific external factors to their management that could have disrupted sleep (i.e. parental alarms) and therefor there could have been other factors outside of the scope of the study influencing sleep quality. When analysing the subjective sleep questionnaires, it is important to note that the parent completing the surveys may not have been the only parent responsible for administration of the overnight feeds and therefor may not fully understand their child's sleep habits.

Further research is needed to explore the long‐term effects of different nocturnal feed strategies on sleep quality. Our intention is that this preliminary report will lead to larger multi‐centre studies, that will address the current lack of literature on sleep quality in children with hepatic GSDs. The use of CGM in our study provided valuable insights into the nocturnal glycaemic profiles of children with hepatic GSD. Longitudinal studies could assess whether stability of overnight glucose levels could ameliorate sleep disturbances in children with hepatic GSD. The use of CGM in our study provided valuable insights into the nocturnal glycaemic profiles of children with hepatic GSD. Advances and increased utilisation of CGMs in this cohort could allow for adjustment to management strategies to avoid excessive hyperglycaemia noted within this study and enhance our understanding of the relationship between glycaemic control and sleep quality. The presence of poor sleep quality within this cohort and the highlights the importance of integrating regular sleep assessment into standard care for children with hepatic GSD.

This cohort of children diagnosed with hepatic GSD all recorded insufficient sleep duration. This was a result of elevated nocturnal wake periods, which was most pronounced in the two individuals who received OBFs. Most participants also reported clinically significant daytime sleepiness and poor QOL outcomes. Overall, there was poor objective and subjective sleep quality within this paediatric hepatic GSD cohort. This finding has important clinical implications, because even modest sleep restriction in school‐age children impacts daytime function including behaviour and emotional regulation.^37^, ^38^

## AUTHOR CONTRIBUTIONS

MV, MW, AMA, HP and RH all contributed to project concept and study design. LA, HP and RH undertook patient recruitment from Metabolic Medicine clinics. LA, MV and AMA analysed and interpreted actigraphy and sleep questionnaire data. LA and MW analysed and interpreted CGM data. LA was the lead writer of the manuscript. MV, MW, AMA, HP and RH assisted by revising the manuscript during the drafting process. All authors have read and approved the final version of the manuscript.

## FUNDING INFORMATION

Funding provided from Australasian Society for Inborn Errors of Metabolism (ASIEM) small grants.

## CONFLICT OF INTEREST STATEMENT

Lucas Agnoletto, Moya Vandeleur, Mary White, Anne‐Marie Adams, Rebecca Halligan and Heidi Peters all declare they have no conflicts of interest.

## ETHICS STATEMENT

Approval obtained from Human Research Ethics Committee (HREC 85857) at the Royal Children's Hospital.

## PATIENT CONSENT STATEMENT

Written informed consent was obtained from all participants' parents/legal guardians.

## INFORMED CONSENT STATEMENT

All procedures followed were in accordance with the ethical standards of the responsible committee on human experimentation (institutional and national) and with the Helsinki Declaration of 1975, as revised in 2000 (5). Informed consent was obtained from all patients for being included in the study.

## Supporting information


**Appendix S1:** Supporting information.

## Data Availability

The data that supports the findings of this study are available in Table 1 of the manuscript.
