# Correction to “Sleep Quality in Children With Hepatic Glycogen Storage Diseases, a Prospective Observational Pilot Study”

**DOI:** 10.1002/jmd2.70004

**Published:** 2025-02-21

L. Agnoletto, M. Vandeleur, M. White, A‐M. Adams, R. Halligan, H. Peters, “Sleep Quality in Children With Hepatic Glycogen Storage Diseases, a Prospective Observational Pilot Study,” *JIMD Reports* 66, no. 1 (2025): e12462, https://doi.org/10.1002/jmd2.12462.

In the article, there should be a note saying “Rebecca Halligan and Heidi Peters have contributed equally to this work and are co‐last authors”.

We apologize for this error.

