# Supplementary material for: Sleep quality in children with hepatic glycogen storage diseases, a prospective observational pilot study
Source: JIMD Rep. 2024 Dec 10;66(1):e12462. doi: 10.1002/jmd2.12462 (PMC11667772; doi:10.1002/jmd2.12462)
Supplement: Supplementary file 1 — Appendix S1: Supporting information. [file JMD2-66-e12462-s001.docx]

**Table 2. Showing Sleep Scoring Data and Continuous Glucose Monitor (CGM) Subcutaneous Glucose (SG) Summary Statistics**

| Participant Number: | Mean TST [30] | SOL (mins)  (45) | WASO (mins)  (41) | SE (%)  (74) | MFI (%+%)  (26.8) | | PDSS | SDSC | OSA-18 | ASHS | CSHS | Peds-QL | Mean SG (mmol/L) | SD SG (mmol/L) | Time SG < 3.5 mmol/L (%) | Time SG > 10.0 mmol/L (%) |
| --- | --- | --- | --- | --- | --- | --- | --- | --- | --- | --- | --- | --- | --- | --- | --- | --- |
| 1 | 389.3  {720} | 78.1 | 75.1 | 60.3 | 28.0 |  | |  | 28 |  |  |  | 4.88 | 0.80 | 0.62 | 0 |
| 2 | 426.7  {480} | 17.3 | 49.3 | 82.8 | 25.1 | 14 | | 29 | 20 | 2.4 |  | 77.2 | 5.47 | 1.39 | 10.64 | 0 |
| 3 | 512.2  {660} | 6.2 | 75.2 | 75.2 | 47.9 |  | |  | 38 |  | 2.6 | 84.5 | 7.41 | 1.37 | 0 | 3.25 |
| 4 | 441.4  {480} | 17.4 | 29.4 | 81.9 | 10.7 | 22 | | 39 | 21 | 2.2 |  | 89.1 | 5.36 | 0.82 | 0 | 0 |
| 5 | 390.7  {480} | 25.1 | 38.1 | 79.9 | 22.8 | 21 | | 33 | 22 | 2.3 |  | 73.9 | **8.65** | 1.40 | 0 | 16.48 |
| 6 | 459.0  {540} | 11.6 | 50.4 | 80.4 | 33.9 | 21 | | 40 | 31 |  | 2.4 | 79.4 | 6.77 | 1.30 | 0.25 | 0.63 |
| 7 | 436.9  {540} | 6.4 | 68.0 | 84.5 | 30.4 | 17 | | 37 | 27 | 2.3 |  | 84.8 | 6.32 | 0.94 | 0.55 | 0 |

**Note:** TST: Total sleep time. SOL: Sleep onset latency. WASO: wake after sleep onset. Average No. awakenings: Number of awakenings per night. SE: Sleep efficiency. MFI: Movement Fragmentation Index. CGM: Continuous Glucose Monitor. SD: Standard Deviation. SG: Subcutaneous Glucose. PDSS: Paediatric Daytime Sleepiness Scale. SDSC: Sleep Disturbance Scale for Children. ASHS: Adolescent's Sleep Hygiene Scale. CSHS: Children’s Sleep Hygiene Scale. Peds-QL: Paediatric Quality of Life Inventory 4.0 Generic Core Scale. Numbers in (brackets) indicate clinically significant cut-off scores used for actigraphy (22, 23). Time in {brackets} shows minimum recommended TST based on their age. Underlined numbers represent values outside the normal range for their respective measure. Bold and underlined numbers represent mean BG levels that fall outside the recommended range (4.8 to 7.0 mmol/L)
